# Understanding how environmental factors influence reproductive aspects of wild myomorphic and hystricomorphic rodents

**DOI:** 10.1590/1984-3143-AR2020-0213

**Published:** 2021-03-30

**Authors:** Maiko Roberto Tavares Dantas, João Batista Freire Souza-Junior, Thibério de Souza Castelo, Arthur Emannuel de Araújo Lago, Alexandre Rodrigues Silva

**Affiliations:** 1 Laboratório de Conservação de Germoplasma Animal, Universidade Federal Rural do Semi-Árido - UFERSA, Mossoró, RN, Brasil; 2 Laboratório de Biometeorologia e Biofísica Ambiental, Universidade Federal Rural do Semi-Árido - UFERSA, Mossoró, RN, Brasil

**Keywords:** climate, seasonality, reproductive physiology, Rodentia, wildlife

## Abstract

Myomorphic and hystricomorphic rodents are vital for maintaining various ecosystems around the planet. This review enables a better understanding of how these rodents respond to environmental factors and adapt to climate adversities. Innumerable factors, such as photoperiod, rainfall, and temperature, can impair or contribute to the quality of rodent reproductive parameters. Prolonged animal exposure to high ambient temperatures alters thermoregulation mechanisms and causes testicular and ovarian tissue degeneration and hormonal deregulation. Photoperiod influences the biological circannual rhythm and reproductive cycles of rodents because it strongly regulates melatonin secretion by the pineal gland, which modulates gonadotropic hormone secretion. Rainfall quantity directly regulates the abundance of fruits in an ecosystem, which modulates the reproductive seasonality of species which are most dependent on a seasonal fruit-based diet. Species with a more diversified fruit diet have smaller reproductive seasonality. As such, habitats are chosen by animals for various reasons, including the availability of food, sexual partners, intra-and inter-specific competition, and predation. This knowledge allows us to monitor and establish management plans to aid in conservation strategies for wild rodent species.

## Introduction

According to the International Union for Conservation of Nature (IUCN), animal extinction occurs at a much higher rate than speciation, which is estimated to occur in at least 25% of endangered mammal species (IUCN, 2019). This event is probably a consequence of climate change caused by global warming, which limits the survival of species sensitive to large variations in climate elements. Anthropic actions also exert a strong influence on species extinctions due to habitat destruction by burning, deforestation (Comizzoli et al., 2009; Comizzoli, 2015), and predatory hunting (Bodmer et al., 2018; El Bizri et al., 2018). These factors may induce the disappearance of a single species in an equilibrated ecosystem, which can compromise the functioning of an entire food chain, harming several directly or indirectly involved populations.

The largest number of placental mammalian species belong to the rodent order, with over 2,000 cataloged species, accounting for a total of 40% of the species in the mammal class, inhabiting all continents and islands, except Antarctica (Carleton and Musser, 2005). The importance of rodents is enormous, as they are vital to the maintenance of many ecosystems around the world because these animals reproduce quickly and are part of the diet of various predatory species (Muñoz et al., 2009). In addition, rodents such as those belonging to Myomorpha and Hystricomorpha suborders act as excellent seed dispersers in various biomes (Muñoz and Bonal, 2011).

Various environmental factors influence reproduction in rodents, including photoperiod (Muteka et al., 2006c; Trillmich et al., 2009; Tavolaro et al., 2015), rainfall (Dubost et al., 2005; Sarli et al., 2016; El Bizri et al., 2018), and temperature (Sarli et al., 2016; Salman et al., 2017; Fabio-Braga and Klein, 2018) Understanding how these environmental factors affect rodents is essential for identifying the physiological and behavioral responses of these animals, thus making it possible to establish strategies that can mitigate the deleterious effects on reproductive activity (Rezende and Bozinovic, 2019), whether caused by climate change, or direct anthropic actions that harm ecosystems.

Studies on the reproductive characteristics of wildlife animals are more complex than those of farm and domestic animals, and for many species, the literature is still scarce. To fill the gaps in these characteristics, many wild species are bred in captivity, either for conservation, preservation, the establishment of biotechnology protocols, or to study their biology (Frankham, 2008; Comizzoli et al., 2009; Comizzoli, 2015; Queiroz et al., 2020). In this context, studies of captive wild rodents may become an important strategy for their conservation (Praxedes et al., 2018), particularly if the rodent species show good acclimatization to captivity and relatively easy management (Slade et al., 2014; Castelo et al., 2015; Queiroz et al., 2019). This practice allows us to understand more efficiently how environmental variables influence the characteristics and behavior of rodents, thus improving our understanding of their reproductive biology.

This review addresses the different responses of reproductive aspects of wild rodents of the suborders Myomorpha and Hystricomorpha to the climatic elements of different biomes, highlighting how the main abiotic factors can affect their reproduction in different weather conditions, whether as free-living animals or bred in captivity. This information could assist in the monitoring of these animals by providing support for the improvement of management strategies and assisted reproductive techniques aimed at their preservation in the face of climate adversity.

## Myomorphic and hystricomorphic rodents – general aspects

Myomorpha is the largest suborder of rodents, containing around 1130 species, almost a quarter of all mammalian species (Carleton and Musser, 2005). This group includes the superfamilies Muroidea (bamboo rats, hamsters, true rats, true mice, gerbils, spiny dormice, lemmings, and voles) and Dipodoidea (jerboas and jumping mice). They are classified according to the structure of the mandible and molar teeth, where part of the medial masseter muscles is inserted into the mandible, in addition to crossing the infraorbital foramen to insert into the rostral muzzle (O’Malley, 2005). Myomorphs are cosmopolitan and generally nocturnal granivores.

In a broader sense, the suborder Hystricomorpha refers to rodents with a hystricomorphous zygomasseteric system, being medium to large rodents. This large group includes the superfamily Ctenodactylidae (comb rats), and the infraorders Caviomorpha (agoutis, pacas, capybaras, guinea pigs, spiny rats, chinchillas, and viscachas) and Phiomorpha (dassie rats and mole-rats) (Carleton and Musser, 2005). They are widely distributed in South America and Africa and act as excellent seed dispersers (Muñoz and Bonal, 2011) because they feed on fruits and leaves (Dubost and Henry, 2017).

For the present review, a keyword search for relevant literature presenting data related to the influence of abiotic factors, such as photoperiod, rainfall, and temperature, on rodent reproduction was conducted, focusing on the suborders Myomorpha and Hystricomorpha. For the search, we used PubMed, Science Direct, Google Scholar, Web of Science, and SCOPUS. Date limitations were removed, but Boolean terms “AND” and “OR” were included wherever possible. Additional relevant articles were sought from the reference lists of all included studies using the snowball sampling method. The authors independently screened the manuscripts to reduce bias and improve the reliability of the findings.

## Photoperiod

The period of daylight hours in a given geographic region, represented by day length, is called the photoperiod (Silva, 2000). Its length depends on latitude and season, with greater variation the further away from the equator. The ability of animals to react to the photoperiod duration is called photoperiodism, which can affect its behavior and reproduction (Santos, 2002; Muteka et al., 2006c; Tavolaro et al., 2015).

**Figure 1:**
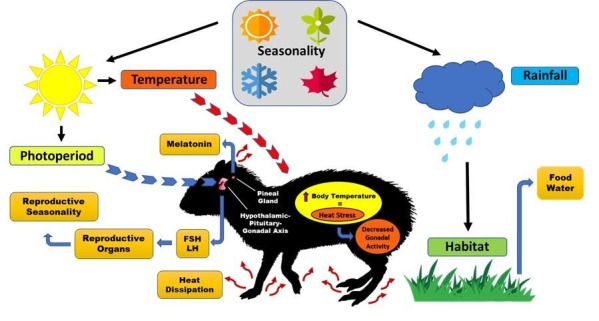
Schematic design of how the main environmental elements influence the reproduction of rodents. In general, the more distant a region is from the equator, the greater the variations in temperature and rainfall between the seasons, thus representing the seasonality. Moreover, photoperiod is related to the presence/absence of light that causes variations in melatonin production by the pineal gland, which modulates the FSH and LH levels, resulting in the regulation of gonadal activity and reproductive seasonality. Rainfall refers to the precipitation that is intricately linked to the supply of water and food, which in some cases may cease the reproductive cycle if the habitat does not have an abundance and variety of resources throughout the year. Finally, the excessive and prolonged heat stress tends to increase body temperature, which decreases gonadal activity; however, some rodents can dissipate this heat through body regions that are usually rich in blood vessels and low in hair, such as the ears, paws, and tail.

The photoperiod duration (Figure 1) is one of the most important drivers of the biological circannual rhythm of an animal (Table 1), because it strongly regulates melatonin secretion by the pineal gland (Tavolaro et al., 2015; Gallol et al., 2020). Melatonin is a neurotransmitter responsible for informing the body of the daylight hours and corresponding time of year (Rocha et al., 2013). It modulates the secretion of follicle stimulating hormone (FSH) and luteinizing hormone (LH) through membrane receptors in hypothalamic-pituitary-gonadal axis cells, regulating the seasonal rhythms and reproductive cycles of several mammals, including rodents (Muteka et al., 2006c; Tavolaro et al., 2015). In viscachas (*Lagostomus maximus*), which are rodents of two genera (*Lagidium* and *Lagostomus*) in the family Chinchillidae, native to South America, it is well demonstrated that melatonin variation strongly affects endocrine physiology, thus causing photoperiod-dependent seasonality in both males (Acosta and Mohamed, 2011) and females (Busolini et al., 2017).

**Table 1:** Influence of different climatic factors on reproductive aspects of rodent species.

**Species**	**Gender**	**Abiotic factor**	**Geographic location**	**Main outcomes**	**Authors**
*Aethomys ineptus*	Male and female	Photoperiod	South Africa	This is a reproductive seasonal species restricted to the summer and fall months, in the south hemisphere.	Muteka et al. (2006a)
*Aethomys namaquensis*	Male and female	Photoperiod	Southern Africa	Pregnant and lactating females were observed only during spring and summer, besides a significant increase in testicular volume, seminiferous tubule diameter and testosterone plasma concentration in males.	Muteka et al. (2006b)
*Aethomys ineptus*	Male	Photoperiod	South Africa	Both species exhibited significantly higher testicular mass when exposed to high photoperiod than during short light hours.	Muteka et al. (2006c)
*Aethomys namaquensis*					
*Acomys dimidiatus*	Male and female	Rainfall and temperature	Saudi Arabia	This animal is a seasonal breeder that can breed opportunistically. Male and female correlate reproductive recrudescence to rainfall. Pregnancies occur in most seasons apart from the winter.	Sarli et al. (2016)
*C57BL/6J mice*	Male	Temperature	Under laboratory conditions	Heat caused by high temperature cause damage to the testicles, such as testicular atrophy, presence of vacuolization and perforations in the seminiferous tubule epithelium, germ cell death.	Zhang et al. (2020)
*Cavia aperea*	Female	Photoperiod	Under laboratory conditions	Female pups kept in groups of two matured at about 47 days when born into lengthening and 79 days when born into shortening day length. They kept under identical short-day conditions after weaning on day 20 of life.	Trillmich et al. (2009)
*Cuniculus paca*	Female	Rainfall	Amazon rainforest	The precipitation of upland forest fruiting was positively correlated with precipitation, which was causally related to higher rates of pregnancy, lactation, and weaning of offspring.	El Bizri et al. (2018)
*Fischer 344 rat (F344)*	Male	Photoperiod	Under laboratory conditions	Rats held under photoperiods of ≥ 12 h of light/day showed increased growth, food intake and higher paired testes weight relative to rats held under photoperiods of ≤ 10 h of light/day.	Tavolaro et al. (2015)
*Gerbilliscus leucogaster*	Male and female	Rainfall and temperature	Namibia	The ovarian activity increased at the end of the dry period and throughout the wet months. During the wet months, pregnant and lactating females were found, besides a increase of testicular mass relative to body mass, testicular volume, and seminiferous tubule diameter in males.	Muteka et al. (2018)
*Lagostomus maximus maximus*	Male	Reproductive seasonality	Argentina	Greater number of morphological defects was observed in the period of decreased gonadal activity (33.8%, winter) than in the activity period (7.8%, summer-autumn). The morphological characteristics of sperm undergo significant changes during their reproductive cycle.	Cruceño et al. (2013)
*Lasiopodomys brandtii*	Male	Photoperiod	China	It was displayed a synchronous peak in gonadal activity with annual day length around summer solstice. The hypothalamic photoperiod genes studied regulate seasonal breeding in a natural rodent population.	Wang et al. (2019)
*Myoprocta exilis, Dasyprocta leporina, Cuniculus paca*	Male and female	Rainfall and temperature	French Guiana forest	The species tended to breed in the period corresponding to the largest supply of fruits from their diets. It was linked to the seasonal importance of fruits in diets, the most aseasonal species having the most diversified diet during the poor fruit season.	Dubost et al. (2005)
*Wistar rat*	Female	Temperature	Under laboratory conditions	The high temperature is responsible for suppression of ovarian function by decreasing the expression of steroidogenic enzymes, estrogen and gonadotropin receptors in the ovary.	Zheng et al. (2019)

In equatorial regions, which have little difference in daylight length throughout the year, animals are minimally influenced by this phenomenon. In contrast, in regions closer to the tropics, where photoperiod fluctuation has a greater influence on animals, reproductive seasonality is more evident, and is also related to thermal stress and nutritional deficiencies depending on the time of year (Muteka et al., 2006a, b, c).

Photoperiod can influence the onset of puberty in female rodents, as demonstrated by Trillmich et al. (2009) in guinea pigs (*Cavia aperea*) (Rodentia, Caviidae) under laboratory conditions. The study showed that individuals that were exposed to many hours of light per day (14:10 h, light:dark), reached puberty within 47 days, while under the lowest light exposure per day, individuals reached puberty in 79 days (10:14 h, light:dark).

In males, the influence of photoperiod on various reproductive aspects is also evident. Studying the Tete veld rat (*Aethomys ineptus)* and the Nomaq rock rat *(Aethomys namaquensis*), Muteka et al. (2006c) demonstrated that during long days (16 h photoperiod), both species exhibited significantly greater testicular mass in relation to body mass, in addition to greater testicular volume and seminiferous tubule diameter, than during short days (8 h photoperiod). However, it is necessary to emphasize, that different rodent species can present different responses to photoperiod, since only in *A. namaquensis*, circulating plasma testosterone concentrations showed higher values on long days (Muteka et al., 2006c). In addition, Wang et al. (2019) onducted an interesting study, which demonstrated that hypothalamic photoperiod genes (*Dio2/3*, *Rfrp-3*, *Kiss-1*, and *GnRH*) regulate seasonal breeding in a natural wild rodent population of male Brandt's voles (*Lasiopodomys brandtii*) from inner Mongolia, China. Over the four years of study, the researchers verified that these rodents exhibited a synchronous peak in photoperiod-related gonadal activity around the summer solstice, as evidenced by the high expression of hypothalamic genes and better reproductive parameters in these periods. It was observed that testicular mass, epididymis mass, and fecal testosterone levels were positively correlated with day length, with the best values obtained between June and July (summer).

## Rainfall

The rain precipitation quantity (Figure 1) for a region in a given time is called rainfall (Silva, 2000). Unpredictable rain and large variations in air temperature influence the reproduction of small mammals (Table 1). Harsh conditions associated with arid environments, where energy and water are severely restricted, may limit or even cease reproduction in rodents during the favorable period.

Sarli et al. (2016) observed that the free-living rat *Acomys imidiatus* found in Saudi Arabia reproduces seasonally, stopping reproduction during the dry season, which corresponds to autumn and winter in that region. This is closely linked to the rainfall quantity and indirectly to other factors such as salinity in the vegetation of this desert, which affects the availability of food for these rodents (Figure 1). The researchers also found that the number of ovarian follicles in the females was significantly lower during the dry autumn and winter (0 mm rainfall) than during the spring and summer rainy season (61.8 mm rainfall). In males, testicular volume and seminiferous tubule diameter were also significantly higher during spring and summer. Plasma testosterone concentrations in males and progesterone in females were also significantly higher during the rainy (summer) period than during the dry period (Sarli et al., 2016).

Similar results were found by Dubost et al. (2005), who demonstrated an improvement in gestation rates in three free-living wild rodent species, the acouchi (*Myoprocta exilis)*, paca (*Cuniculus paca)*, and crown-rumped agouti (*Dasyprocta leporina)*, during increased rainfall in the French Guiana rainforest. The researchers attributed the reproductive seasonality in these species to the production of some forest fruits, which are important in the diet of these animals (Figure 1). These fruits depended directly on the rainfall amount to regulate their abundance, and thus, the less seasonal species had a more diverse diet during the scarcer fruit season.

For tropical regions, in a 15-year participatory study in two areas in the northwest of the Amazon, El Bizri et al. (2018) analyzed reproductive organs of pacas (*C. paca*) obtained from voluntary donations by hunters. They used data on precipitation, river water level, and fruiting phenology. They concluded that the upland forest fruiting was positively correlated with precipitation (Figure 1), which was directly linked to higher rates of pregnancy, lactation, and weaning of offspring.

## Temperature

The study of animal thermal performance curves is generally used to anticipate the effects of ambient temperature on characteristics of interest in these organisms and is one of the means to predict the potential effects of global warming on ecological systems (Rezende and Bozinovic, 2019), since ambient temperature (Table 1) is essential for maintaining animal health (Silva, 2000; Santos, 2002).

Sudden changes in temperature cause heat stress, usually causing immunological depreciation, which makes animals more susceptible to infections (Silva, 2000). Homeothermic animals, such as rodents, are known to respond to environmental thermal variations by modulating thermogenesis, thereby activating sensible and evaporative heat transfer mechanisms, either for loss or gain of thermal energy (El-Sabrout, 2018; Mascarenhas et al., 2018). In some rodent species, such as the mole rats (*Fukomys mechowii* and *Heliophobius argenteocinereus*) (Šumbera et al., 2007) and agoutis (*Dasyprocta aguti*) (Queiroz et al., 2019), there is a physiological pathway for heat dissipation through a body area that is normally rich in blood vessels and low in hair, that facilitates heat dissipation (Figure 1); this is called the thermal window (Romanovsky et al., 2002). In this regard, Queiroz et al. (2020), studying Spix's yellow-toothed cavy (*Galea spixii*) in a semi-arid environment in northeastern Brazil, recently identified that the pinnae and vibrissae regions act as thermal windows, thus being the first line of defense against overheating.

Most small rodents have a thermal comfort range of 21-24 °C, while laboratory animals such as guinea pigs adapt better to the temperature range of 18-20 °C (Santos, 2002). Prolonged exposure to high temperatures alters thermoregulatory mechanisms, rendering them inefficient in dissipating excess body heat (Rashamol et al., 2018; Maurya et al., 2019), which is the main cause of infertility in male animals (Setchell, 2006). In fact, some free-living African wild rodents cease reproductive activities during periods of high temperatures, as observed for *A. ineptus* (Muteka et al., 2006a), *A. namaquensis* (Muteka et al., 2006b), and *Gerbilliscus leucogaster* (Muteka et al., 2018). In addition, as demonstrated under laboratory conditions, exposure to low temperatures causes respiratory problems (Santos, 2002) and reduces basal metabolism in free-living rodent species (Muteka et al., 2018), thus indirectly decreasing their reproductive potential.

Depending on the intensity of the thermal injury, testicular parenchyma degeneration and serious damage to spermatogenesis may occur (Kleisner et al., 2010; Durairajanayagam et al., 2014; Fabio-Braga and Klein, 2018). To illustrate this, Zhang et al. (2020) subjected male C57BL/6J mice to two overheating treatments at 39 and 42 ºC, submerging the lower parts of the body (hind legs, tail, and scrotum) in a thermostatically controlled water bath for 20 min. They observed that heat treatment at 42 ºC triggered the greatest damage to the testicles, such as testicular atrophy, presence of vacuolization and perforations in the seminiferous tubule epithelium, germ cell death, and fracture of the sterile junction of Sertoli cells. However, the treatment at 39 ºC had no significant impact on testicular histology and germ cell morphology, which suggests the existence of a threshold of the testicular response to thermal stress at this temperature.

The degeneration of ovarian tissue and reduction in folliculogenesis and oogenesis were also observed as responses to thermal injury in females (Zheng et al., 2019). In prepubertal female Wistar rats, Zheng et al. (2019) observed the ovarian function of these animals in the face of thermal stress at different temperatures (38, 40, and 42 °C for 2 h/ day). The plasma levels of LH and triglycerides and the expression of LH receptors FSH and estradiol-17β in the ovaries were significantly lower at higher temperatures (especially at 42 °C) than in the control (26 °C). Liver metabolic function was significantly reduced in rats heated to 42 °C, as caspase-3 and NK-κB gene expression were higher at higher temperatures. These results indicate that high temperatures may suppress ovarian function, decreasing the expression of steroidogenic enzymes, estrogen receptors, and gonadotropin in the ovary (Figure 1).

It is important to note, however, that depending on the species, the higher temperature will not always negatively affect reproduction. For example, when studying free-living Saudi Arabian rats (*A. imidiatus*), Sarli et al. (2016) found that during the months with the highest average air temperature (spring and summer), the best reproductive parameter results in the testes and ovaries occurred, along with higher values of testosterone and plasma progesterone in males and females, respectively. This was influenced by another element closely associated with the period of the year, rainfall, as previously mentioned. It is therefore clear that sometimes one isolated abiotic factor is not sufficient to influence reproductive performance of some species, but instead a sum of various factors that characterize the habitat in which the individuals live.

## Habitat

The climate of a region is established by a series of variable atmospheric conditions throughout the year, such as rainfall, photoperiod, humidity, and air temperature (Bronson and Heideman, 1994). In each biome, these meteorological variables (Figure 1) directly or indirectly affect the fauna, flora, and relief. These geophysical interactions cause, in addition to the biotic factors, fluctuations in food and water availability during the year, by the environment (Stapp, 1997). This defines the survival of certain animal populations in a given habitat, and restricts or eliminates the reproduction and presence of those most sensitive to fluctuations in these resources (Wube et al., 2009; Muteka et al., 2006c, 2018). Habitats are chosen by animals for various reasons, mainly the availability of food, sexual partners, intra- and inter-specific competition and predation.

According to Stapp (1997), there are several survival criteria for the choice of habitat. For instance, insectivorous rodents that inhabit the state of Colorado, United States, choose microhabitats based on the availability of prey, according to seasonal and spatial variations and other resources such as water (Stapp, 1997), as illustrated in Figure 1. In contrast, pacas (*C. paca*) from the Atlantic Forest in southern Brazil chose their microhabitat based on good water availability and the presence of a dense forest cover (Goulart et al., 2009). According to these researchers, information on habitat selection allows more efforts to be directed to the habitat associated with focal species, and indicates the importance of environmental heterogeneity, which makes species coexistence possible.

In a continental country such as Brazil, small mammals may be subjected to different habitats and respond accordingly. In the Atlantic Forest, a region on the Brazilian east coast, where there is a predominance of dense forests, wetlands, and great diversity of fauna, Gentile and Fernandez (1999) observed that different species of Sigmodontinae rodents (*Nectomys squamipes*, *Akodon cursor*, and *Oligoryzomys nigripes*) respond to different aspects of the microhabitat, thus presenting significant oscillations in their populations throughout the year. In the Cerrado biome, in midwestern Brazil, Santos-Filho et al. (2012) found no relationship between microhabitat, richness variables, and abundance of small rodent populations. In Pantanal (another Brazilian biome), Santos-Filho et al. (2008) analyzed 13 rodent species captured in forest fragments of the region and found no significant variation in the total richness and abundance of these animals between the dry and rainy periods of the year.

It is necessary to highlight, however, that sometimes, even among animals that coexist in the same habitat, large differences occur between genetically close rodent species. These aspects were clearly described by Dubost et al. (2005) in their study on rainforest rodents from French Guiana (*M. exilis, C. paca,* and *D. leporina*), which showed reproductive differences among them, regarding sexual behavior, puberty, and reproductive seasonality. It is therefore evident that the survival of a species is a consequence of the combination of its ability to successfully reproduce and minimize the loss of offspring through adaptation in its own microenvironment, which eventually has a direct influence on the choice of most suitable habitat for each species.

## Reproductive seasonality

Reproductive seasonality is the phenomenon by which some species decrease or cease sexual activity during a certain period of the year, usually caused by climatic factors, especially temperature, rainfall, or photoperiod (Henry and Dubost, 2012; Maia and Silva, 2016). It is known that the effects of seasonality can cause changes in both the morphophysiology and biochemistry of sexual gonads during the reproductive cycle (Aguilera-Merlo et al., 2009).

Female rodents may exhibit one or more series of estrous cycles during the reproductive season, directing the parturition to the most favorable period for reproduction (Bronson and Heideman, 1994; Henry and Dubost, 2012; Muteka et al., 2006a, b, c). In contrast, some species may present a non-seasonal reproduction as observed for female black agoutis (*Dasyprocta fuliginosa*) that present a non-seasonal polyestrous cycle and can reproduce throughout the year (Mayor et al., 2011).

Male rodents can increase the testicular volume and seminiferous tubule diameter, thus improving spermatogenesis efficiency during reproductive seasons (Muteka et al., 2018). This variation in the activity of the gonads as a response to different seasonal periods suggests a possible evolutionary strategy for improved opportunistic breeding (Bronson and Heideman, 1994; Muteka et al., 2006c, 2018), which focuses on the availability of resources such as food, water, and favorable environmental conditions.

To exemplify the role of seasonality, we highlight the study on Namaqua rock rats (*A. namaquensis*) conducted by Muteka et al. (2006b) in South Africa. The researchers observed the occurrence of pregnant and lactating females only during spring and summer, while no lactating females were registered during autumn and winter. Gonadal activity was determined by the evaluation of ovarian follicles, corpora lutea, progesterone, and 17b-estradiol plasma concentrations, and was significantly higher during spring and summer, compared with autumn and winter. In males, plasma testosterone concentration, testicular volume, and seminiferous tubule diameter increased significantly during spring and summer, while low to no spermatogenesis or presence of sperm in the epididymis was observed in autumn and winter.

The effects of rainfall and fruit diet on the male and female reproductive characteristics were also observed for the acouchi (*M. exilis*), paca (*C. paca*), agouti (*D. leporina*), and spiny rat (*Proechimys cuvieri*), raised in the French Guiana rainforest. All the species presented slightly pronounced reproductive seasonality and synchronism regarding birth, directing them to the rainiest months with a greater variety of fruit (November to April). The researchers found that the more the diets of species were composed of fruits, the more seasonal were the births (Dubost and Henry, 2017).

It is worth noting that the reproductive seasonality of wild rodents is modulated by environmental variables and their joint interactions. In regions where rainfall is scarce, such as in deserts or semi-arid climates, high temperatures and low air humidity are observed throughout the year (Sarli et al., 2016; Salman et al., 2017), which can promote serious energy and water restrictions that may affect the reproduction of small mammals. Despite this, some rodent species as free-living Saudi Arabian rats (*A. imidiatus*) are largely acclimated to arid conditions by adapting their reproductive cycle to environmental conditions (Sarli et al., 2016).

When studying male viscachas (*L. maximus*) in Argentina, Cruceño et al. (2013) aimed to relate the morphological changes in epidydimal sperm evaluated through scanning electron microscopy (SEM) with the period of the year of full (summer-autumn) or reduced (winter) gonadal activity. The analysis revealed that the sperm from the epidydimal corpus and cauda had great seasonal variations in structural parameters. In general, a greater number of morphological defects were observed in the period of decreased gonadal activity (33.8%) than in the activity period (7.8%). These outcomes confirm that the morphological characteristics of *Lagostomus* sperm undergo significant changes during their reproductive cycle under a seasonal influence.

## Final considerations

Myomorphic and hystricomorphic rodents show physiological changes in response to abiotic factors present in the environment in which they live. Temperature, rainfall, and photoperiod represent the main environmental agents that regulate, interfere, or contribute to their reproductive capacity. Temperature is the main element that can impair reproduction in most species, but the combined effect of other climate variables cannot be disassociated from temperature, which can increase reproduction losses or mitigate the damage caused by it. Some species of wild rodents suffer less effects caused by a specific climate element, while others, even belonging to the same habitat, show great changes in their reproductive parameters due to variations of this element throughout the year. These differences fluctuate according to the supply and competition for food and water resources, sexual partners, and predation, with aim to achieve success in species survival and opportunistic breeding.
